# Acute Pancreatitis Severity Prediction: It Is Time to Use Artificial Intelligence

**DOI:** 10.3390/jcm12010290

**Published:** 2022-12-30

**Authors:** Dorottya Tarján, Péter Hegyi

**Affiliations:** 1 Heart and Vascular Center, Division of Pancreatic Diseases, Semmelweis University, 1083 Budapest, Hungary; 2 Centre for Translational Medicine, Semmelweis University, 1085 Budapest, Hungary; 3 Institute for Translational Medicine, Medical School, University of Pécs, 7623 Pécs, Hungary; 4 Translational Pancreatology Research Group, Interdisciplinary Centre of Excellence for Research Development and Innovation University of Szeged, 6725 Szeged, Hungary

**Keywords:** machine learning, albumin, CRP, WBC, BISAP, mortality, severity

## Abstract

The clinical course of acute pancreatitis (AP) can be variable depending on the severity of the disease, and it is crucial to predict the probability of organ failure to initiate early adequate treatment and management. Therefore, possible high-risk patients should be admitted to a high-dependence unit. For risk assessment, we have three options: (1) There are univariate biochemical markers for predicting severe AP. One of their main characteristics is that the absence or excess of these factors affects the outcome of AP in a dose-dependent manner. Unfortunately, all of these parameters have low accuracy; therefore, they cannot be used in clinical settings. (2) Score systems have been developed to prognosticate severity by using 4–25 factors. They usually require multiple parameters that are not measured on a daily basis, and they often require more than 24 h for completion, resulting in the loss of valuable time. However, these scores can foresee specific organ failure or severity, but they only use dichotomous parameters, resulting in information loss. Therefore, their use in clinical settings is limited. (3) Artificial intelligence can detect the complex nonlinear relationships between multiple biochemical parameters and disease outcomes. We have recently developed the very first easy-to-use tool, EASY-APP, which uses multiple continuous variables that are available at the time of admission. The web-based application does not require all of the parameters for prediction, allowing early and easy use on admission. In the future, prognostic scores should be developed with the help of artificial intelligence to avoid information loss and to provide a more individualized risk assessment.

## 1. Introduction

Acute pancreatitis (AP) is among the most common gastroenterological disorders that frequently present in emergency departments. Most patients only develop mild or moderate AP. However, around 5–10% of patients will progress to severe acute pancreatitis (SAP) in which the mortality ranges from 10% to 50%, in contrast to the overall mortality of 2–5% [1,2].

As the clinical course strongly depends on the early management of AP, predicting the severity of the disease, different organ failure, or infected pancreatic necrosis is of high importance. Recently many single and multiparametric scores have been published to predict the outcome of the disease. Therefore, we felt it important to summarize our current knowledge in the field.

## 2. Univariate Biomarkers

The on-admission levels of C-reactive protein (CRP) and white blood cell count (WBC) were found to be associated with SAP; however, they have a very poor AUC (0.681) [3,4]. Triglyceride (TG) levels have also been shown to dose-dependently predict local complications, respiratory and heart failures, SAP, and mortality [5]. Other metabolic factors, such as glucose, hypertonia, and obesity, were also predictive of SAP [6,7]. Hypoalbuminemia is a good predictive factor for respiratory failure and the local complications that elevate the probability of SAP and mortality [8]. The on-admission signs of renal failure can have predictive potential as well. Elevated BUN or creatinine is also associated with worse outcomes of AP [9]. Haemoconcentration, i.e., an elevated hematocrit level also has a predictive role in the early phase of AP [10]. Not only the laboratory parameters but the anamnestic data, such as a history of alcohol or smoking, have a predictive value as well [11]. Age, comorbidities, and on-admission pain have also shown associations with SAP [12,13,14,15,16]. However, all of these parameters have poor accuracy (AUC 0.5–0.7); therefore, these biomarkers cannot be used in clinical settings alone.

## 3. Multivariate Scores

Score systems have been developed to predict the severity by using 4–25 factors. The Bedside Index of Severity in Acute Pancreatitis (BISAP) was developed to predict early severity and mortality within the first 24 h after admission [1]. The modified computed tomography severity index (mCTSI) score is also an equivalent option to predict severity and mortality; however, it is usually not available at the time of admission [17]. The Acute Physiology and Chronic Health Examination (APACHE) II score was originally created to foretell patients’ outcomes in the intensive care unit; thus, it is not specific to AP [18]. The Ranson and Glasgow score was specifically developed to predict mortality and severity in AP [19]. However, there are two major disadvantages of these score systems: (i) one of them is that they require multiple parameters, not just including the usually measured variables; (ii) secondly, these parameters need to be collected twice within 48 h. Therefore, their usability is also limited.

## 4. Artificial Intelligence (AI)

Artificial intelligence can detect the complex nonlinear relationships between multiple biochemical parameters and disease outcomes. Therefore, it can be used to generate prognoses in the healthcare system. Machine learning is an application of artificial intelligence, and through the use of statistical methods, algorithms are trained to make predictions. It allows a computer system to continue learning and improving on its own based on experience. Artificial intelligence has proven valuable in other fields; for example, in diabetes care or in radiological diagnosis [20,21]. This year, we developed two new scores, NECRO-APP, to predict acute necrotizing pancreatitis (ANP), and EASY-APP, to determine the severity of AP [22,23]. The EASY-APP uses multiple variables that are available at the time of admission. This score’s algorithm constructed a model based on a training dataset that was developed and confirmed by a study of almost 5000 patients from multiple countries. EASY-APP can calculate a risk score between 0 and 1 for severe AP while explaining the prediction of the machine-learning model. The web-based application does not require all of the parameters for prediction, allowing for early and easy use on admission. Of course, providing more parameters to EASY-APP will result in a more accurate prediction of the severity of AP [23].

## 5. Here, We Provide Three AP Cases

**Figure 1:**
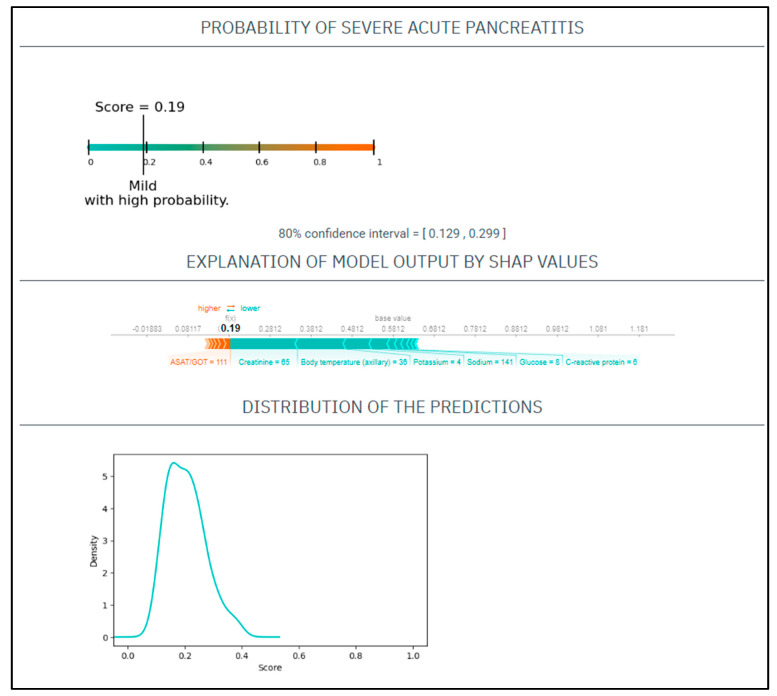
The EASY-APP-s prediction model for the patient in case number 1.

CASE No.1: A 75-year-old woman presents to the emergency department with a 10 h length of epigastric pain. Upon physical examination, she had a heart rate of 85/min and a blood pressure of 142/77 Hgmm, her respiratory rate was 18/min, and she was afebrile. Her laboratory tests on admission revealed a CRP 6 mg/L, WBC 14.5 G/L, amylase 1621 U/L, potassium 4 mmol/L, natrium 141 mmol/L, glucose 8 mmol/L, GOT 111 U/L, BUN 9.5 mmol/L, creatinine 65 umol/L. She had no medical history of smoking or alcohol consumption (Figure 1). 

Based on these parameters her 

EASY score was: 0.19 (CI: 0.129–0.299)

BISAP score was: 2

Finally, the patient had mild AP.

**Figure 2:**
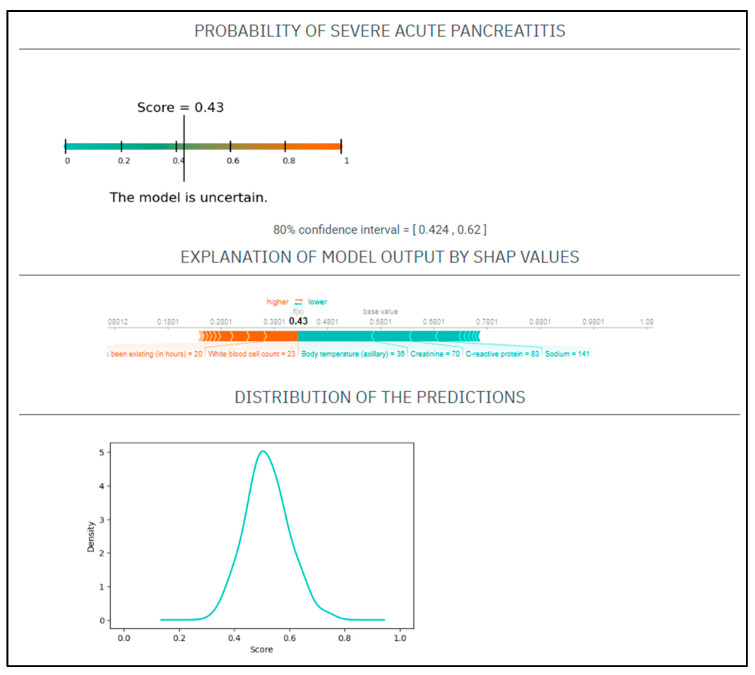
The EASY-APP-s prediction model for the patient in case number two.

CASE No.2: A 71-year-old woman with a history of smoking presented at the emergency department with 20 h onset of abdominal pain. On physical examination, she had a heart rate of 110/min and a blood pressure of 141/76 Hgmm, her respiratory rate was 17/min, and she was afebrile. Laboratory tests showed CRP 83 mg/L, WBC 23 G/L, amylase 1285 U/L, natrium 141 mmol/L, glucose 9.5 mmol/L, GOT 120 U/L, BUN 9 mmol/L, creatinine 70 umol/L (Figure 2).

Based on these parameters her

EASY score was: 0.49 (CI: 0.424–0.62)

BISAP score was: 3

Finally, the patient had mild AP.

**Figure 3:**
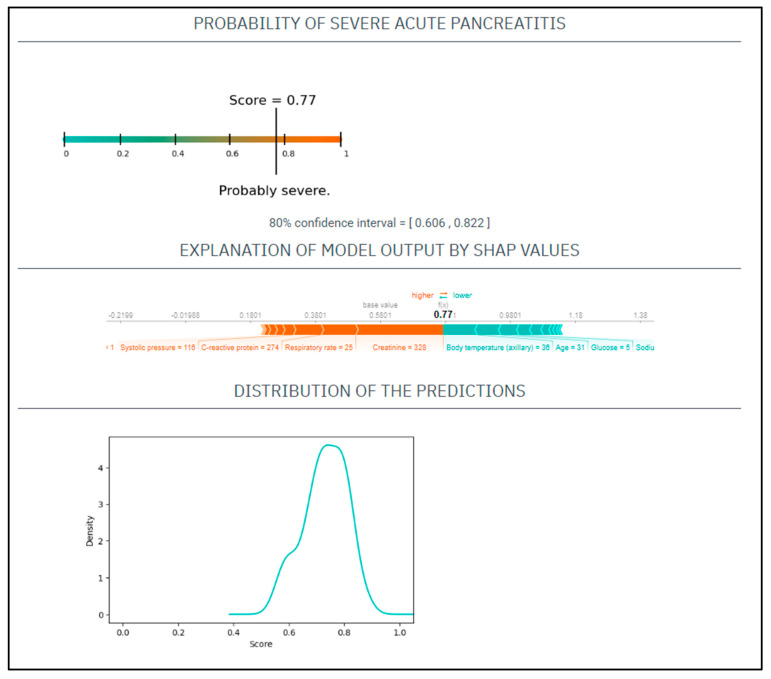
The EASY-APP-s prediction model for the patient in case number three.

CASE No.3: A 31-year-old male with a history of alcohol abuse and smoking presented with severe abdominal pain, which had started 7 days prior. His serum amylase level 943 U/L, CRP 274 mg/L, WBC 13 G/L, natrium 138 mmol/L, glucose 5 mmol/L, GOT 201 U/L, BUN 8.3 mmol/L, creatinine 328 umol/L. On physical examination, he had a heart rate of 135/min and a blood pressure of 116/96 Hgmm, his respiratory rate was 25/min, and he was afebrile (Figure 3).

Based on these parameters his

EASY score was: 0.77 (CI: 0.606–0.822)

BISAP score was: 1.

Finally, the patient had severe AP.

In summary, artificial intelligence has several advantages over the earlier used systems: (i) the prediction value is continuously improving by backloading the severity prediction results, (ii) it is easy to use, (iii) it is not bound to binding parameters, (iv) it also shows the confidence interval of the scoring, (v) there is no lost information (the variables are continuous rather than dichotomous).

## Data Availability

No new data were created or analyzed in this study. Data sharing is not applicable to this article.
